# PIRO and sepsis stratification: reality or a mirage?

**DOI:** 10.5935/0103-507X.20150038

**Published:** 2015

**Authors:** Cristina Granj a, Pedro Póvoa

**Affiliations:** 1 Department of Biomedical Sciences and Medicine, Universidade do Algarve - Faro, Portugal.; 2 School of Medical Sciences, Universidade Nova de Lisboa - Lisbon, Portugal.

## What is stratification?

The objectives of staging systems are to stratify patients with a given disease according to their risk for adverse events, to assess their potential response to a given treatment, and to monitor their actual response. Such systems are widely used, and the Classification of Malignant Tumors (TNM) is the best-known of these systems.^(1)^ In oncology, staging of neoplasms is an essential step in the process of clinical decision-making, as it is crucial for the establishment of the prognosis of the disease and the choice of the most adequate therapeutic approach.^(1)^

The concepts of infection, systemic inflammatory response syndrome (SIRS), sepsis, severe sepsis, and septic shock defined at the American College of Chest Physicians/Society of Critical Care Medicine (ACCP/SCCM) Consensus Conference represented the first attempt at the stratification of sepsis.^(2)^ The fact that the mortality of septic patients increases in parallel to the number of SIRS criteria that they meet was noticed very early, as was its correlation with the presence of organ failure (severe sepsis) and, in particular, septic shock, which exceeded 50% in the original studies.^(3)^

## Second consensus conference and the PIRO concept

Because the results of the application of the definitions formulated in the Consensus Conference held in 1991 did not meet expectations^(4)^ and, in addition, nearly one decade had passed, the Society of Critical Care Medicine, European Society of Intensive Care Medicine, American College of Chest Physicians, American Thoracic Society, and Surgical Infection Society held a second Consensus Conference.^(5)^ On that occasion, it was agreed that the concepts of sepsis, severe sepsis, septic shock, and SIRS were useful, even though the latter has poor specificity despite its high sensitivity. The definition of infection was not changed. Sepsis remained understood as a clinical syndrome defined by both infection and a systemic inflammatory response (sepsis without microbiological documentation can only be classified as a strong suspicion). Those concepts are quite similar to those previously defined by Bone et al.^(2)^ However, it was admitted that those concepts and definitions did not allow for staging or stratifying the risk of septic patients.^(5)^ Thus, the list of signs and symptoms associated with sepsis was increased to more accurately reflect the host’s clinical response to infection.^(5)^

The second Consensus Conference suggested a hypothetical model for stratification of sepsis similar to the TNM model, called “PIRO” (an acronym of Predisposition, Insult, deleterious Response and Organ failure), which was proposed to better describe the sepsis syndrome. The classification is based on four sets of variables: *P*, for *p*redisposition, represents all predisposing factors, comorbidities, and genetic factors present before the occurrence of sepsis; *I,* for *i*nsult or *i*nfection, includes the description of the infection, etiologic agent and its virulence, pattern of sensitivity, localization, and compartment of infection; R, for *r*esponse, considers the type of host response, namely, the inflammatory response and the acute stage response; and O, for *o*rgan dysfunction, corresponds to the number of organs affected and the severity of dysfunction.

PIRO was formulated based on a previous concept, IRO, which was first proposed in 2000 by John Marshall.^(6)^ In addition to a stratification system based on infection (I), host response (R) and organ failure (O), the idea behind IRO was to enable the individualization of treatment. According to this conceptual classification, patients are allocated to one of four possible stages. As an example, stage I (presence of infection with minimal or no systemic response and no organ failure - *I1R0O0*) represents the appropriate population of patients for studies of new antimicrobial agents. In turn, stage IV (presence of a response and severe organ dysfunction - *IxR3O2*) includes appropriate patients for studies of salvage therapies, such as plasmapheresis or the use of immunoglobulin.

## Applicability of PIRO in clinical practice

Although the concept’s construction is attractive from the theoretical point of view, its applicability to actual clinical practice is quite problematic. Several authors have attempted to use the PIRO concept in clinical practice^(7-11)^ but met countless difficulties beginning with the limitations inherent to the studies conducted, such as the studies included only patients with the same diagnosis (community-acquired^(10)^ or ventilator-associated pneumonia),^(11)^ were based on secondary analyses of patients included in other studies with different objectives;^(7,8)^ and included patients for whom the diagnosis of sepsis was based on “suspicion” only, thus allowing the possibility of inclusion of patients without sepsis.^(9)^

## Dynamic approach of the PIRO concept

Granja et al.^(12)^ published an original methodological approach based on a parallel between the dynamic clinical progression of sepsis and a dynamic methodological model for PIRO, rather than assessing the variables corresponding to each PIRO component using static reference values. They evaluated the variation (delta) of the variables included in components I and R using a previously described technique to document the variation of the C-reactive protein levels and also sought to establish how the slope of the curve could have a prognostic capacity.^(13)^ The overall performance of our model was assessed by the area under a receiver operating characteristic curve (AUC-ROC), the value of which was 0.84, i.e., identical or even superior to the methods reported in the previously mentioned studies. Thus, the model was shown to more accurately reflect the initial concept of the need to stratify septic patients to stage the severity of the disease, establish a prognosis, and select the most adequate treatment in a dynamic manner. In short, our approach consists of monitoring the progression of sepsis for the first five days of the disease, in contrast to the classic method, which considers the values at baseline or during the first 24 hours of the disease and produces an evaluation that does not accurately reflect the clinical reality.

Our study had some limitations, including the exclusion of hospital-acquired sepsis; analysis of a single biomarker (C-reactive protein), while other biomarkers or biomarker panels (procalcitonin and cytokines) were excluded; absence of microbiological documentation for approximately 40% of the patients; and non-inclusion of the full range of neoplastic diseases, as neoplasm was defined only as a metastatic disease.

Despite these limitations, the dynamic approach of the PIRO concept is attractive from a clinical point of view, and it can be easily applied at the bedside, as we have shown. The results obtained to date must be validated and tested in other settings and with other cohorts before the approach described here is implemented in clinical practice and its actual value is demonstrated.
